# Computerized acoustic assessment of treatment efficacy of nebulized epinephrine and albuterol in RSV bronchiolitis

**DOI:** 10.1186/1471-2431-7-22

**Published:** 2007-06-02

**Authors:** Raphael Beck, Nael Elias, Shay Shoval, Naveh Tov, Gil Talmon, Simon Godfrey, Lea Bentur

**Affiliations:** 1 Pediatric Pulmonary Unit, Meyer Children's Hospital, Rambam Medical Center, Bruce Rappaport Faculty of Medicine, Technion, Haifa, Israel; 2 Institute of Pulmonology, Hadassah University Hospital, Hebrew University, Jerusalem, Israel

## Abstract

**Aim:**

We evaluated the use of computerized quantification of wheezing and crackles compared to a clinical score in assessing the effect of inhaled albuterol or inhaled epinephrine in infants with RSV bronchiolitis.

**Methods:**

Computerized lung sounds analysis with quantification of wheezing and crackles and a clinical score were used during a double blind, randomized, controlled nebulized treatment pilot study. Infants were randomized to receive a single dose of 1 mgr nebulized l-epinephrine or 2.5 mgr nebulized albuterol. Computerized quantification of wheezing and crackles (PulmoTrack^®^) and a clinical score were performed prior to, 10 minutes post and 30 minutes post treatment. Results were analyzed with Student's t-test for independent samples, Mann-Whitney U test and Wilcoxon test.

**Results:**

15 children received albuterol, 12 received epinephrine. The groups were identical at baseline. Satisfactory lung sounds recording and analysis was achieved in all subjects. There was no significant change in objective quantification of wheezes and crackles or in the total clinical scores either within the groups or between the groups. There was also no difference in oxygen saturation and respiratory distress.

**Conclusion:**

Computerized lung sound analysis is feasible in young infants with RSV bronchiolitis and provides a non-invasive, quantitative measure of wheezing and crackles in these infants.

**Trial registration number**: ClinicalTrials.gov NCT00361452

## Background

Bronchiolitis is the most common cause of hospitalization for respiratory infection in infants under one year of age. Respiratory syncytial virus (RSV) is the most common etiology of acute bronchiolitis in infants. About 1–2% of infants with bronchiolitis need to be hospitalized and approximately 8% of these children require intensive care. Of high risk patients, such as those with bronchopulmonary dysplasia or congenital heart disease, about 30% require intensive care [1-3].

Treatment for most infants with bronchiolitis is usually supportive, including oxygen, hydration and antipyretics. The use of bronchodilator therapy remains controversial [4,5], since the main tool used to measure response (clinical score) is subjective and inaccurate. Thus different studies showed different responses [6-11]. The one study which used an objective measure (airway resistance) showed improvement in only 30% of infants treated with nebulized salbutamol [12]. Similar variable results were reported when the effect of nebulized epinephrine in RSV Bronchiolitis was studied using clinical scores [13-19]. Two studies which measured airway resistance [12,17] were able to demonstrate a significant response to nebulized Epinephrine, which was not apparent by clinical criteria. Thus, objective physiological measurements may be more sensitive and accurate in detecting response to treatment than clinical assessments. The clinical score is a crude instrument for measuring a clinical effect, as it is observer-dependent and may therefore suffer from low objectivity. Pulmonary function tests in infants are objective, but require sedation, which is problematic in acutely ill infants [20]. Wheeze and crackle quantification by lung sounds analysis methods is objective, non invasive and has been shown to correlate with clinical status in asthma and bronchiolitis [21-23]. The recording procedure is simple, requiring only the attachment of 4 ECG-size sensors to the chest wall. A 30-second recording is often adequate, but recording time can be extended as necessary [24], to obtain good quality data, where wheezes and crackles are detected and counted with high degree of accuracy. The recording is, however, susceptible to outside noise interference, and requires a relatively quiet environment during the recording. In addition, the outside-noise elimination algorithm helps in eliminating outside noise from the recording. The instrument is currently expensive, but has the potential of both size and price reduction.

In this study we evaluated the feasibility of using computerized quantification of wheezing and crackles and a clinical score in measuring the effect of a single dose of nebulized albuterol or nebulized epinephrine in infants with RSV bronchiolitis. Our hypothesis was that automated quantification of wheezing and crackles is equal or superior to the clinical score in assessing the infants' response to treatment.

## Methods

### Patients

The study was approved by the Rambam Hospital Ethics Committee. Subjects were recruited from infants treated at the emergency department with proven RSV bronchiolitis over a four-month period. Eligible infants complied with the following: 1) Age 2 – 12 months; 2) First episode of respiratory distress; 3) RSV antigen detected in oropharyngeal secretions by ELISA; 4) Informed consent signed by parents. Infants younger than 2 months and those with chronic lung disease, cardiac disease or other chronic conditions were excluded.

### Study design

Computerized lung sounds recording and analysis with algorithms for wheeze and crackles counts (see below) and a Clinical Score were performed during a double blind, randomized, comparative study, comparing nebulized salbutamol (2.5 mg diluted with 2.5 ml of 0.9% saline) to nebulized l-epinephrine (1 mg diluted with 2 ml of 0.9% saline). Solutions were driven by compressed oxygen of 5 l/min flow (giving a mean output of 0.4 ml/min), and nebulized using a Hudson Up-Draft II nebulizer (Hudson RCI, Temecula, CA, USA). Medication was randomized prior to commencement of the study by the hospital pharmacy independently of trial staff. Randomization was in blocks of 10 (5 salbutamol/5 epinephrine). The two solutions, provided in identical containers, were indistinguishable to the researchers.

### Clinical Assessment

Clinical assessment was done prior to treatment, 10 minutes post and 30 minutes post treatment. At each point a total clinical score was given (Table 1). The total clinical score, modified from Wang et al. [25], was comprised of the following parameters: wheezing, retractions, O_2 _saturation, respiratory rate and heart rate. A score from 0 to 3 was given to each parameter (maximal total score – 15 – indicated severely ill infants). All clinical assessments and scores in all patients were performed by the same investigator for consistency.

**Table 1:** Total clinical score of disease severity in young infants with bronchiolitis.

**Score**	**Wheeze**	**Retractions**	**Oxygen Saturation**	**Respiratory Rate (per min.)**	**Heart rate (per min.)**
0	None	None	≥ 95%	Normal (<35)	< 140
1	Mild	Mild	92 – 94%	35 – 44	140 – 159
2	Moderate	Moderate	90 – 91%	45 – 54	160 – 179
3	Marked	Severe	< 90%	> 55	≥ 180

### Computerized breath sounds recording and analysis

Recording and analysis of respiratory sounds were conducted according to standardized methods previously described [24]. Respiratory acoustic signals were recorded from phonopneumography piezoelectric contact sensors (PPG Sensors, Karmel Medical Acoustic Technologies Ltd., Yokneam Illit, Israel) applied over right and left axillae (AR, AL) and both posterior bases (BR, BL) of the lungs. The sensors are coin-shaped piezoelectric elements with linear ±3dB frequency response from 75 to 2000 Hz, a resonance at 2.7 kHz, a useable range that extends beyond 4 kHz, and a built-in passive ambient noise rejection capability. The sensors were attached to the chest with adhesive foam pads that further reduce ambient noise interference and eliminate contact noise. All sensors were connected to the PulmoTrack^® ^(Model 1010, Karmel Medical Acoustic Technologies Ltd. Yokneam Illit, Israel) where signal conditioning (amplification X3000; band pass filtration 80–4000 Hz at 24 dB/oct) was performed prior to analog-to-digital conversion (12 bit, 11,025 samples per second per channel). Two other signals were tracked: ambient noise – with an air-coupled microphone placed near the patient, and chest impedance for measurement of breathing activity (respiratory rate, phase and amplitude). Wheeze detection was performed by a fast Fourier transform (FFT)-based algorithm that was previously verified and found to have sensitivity of 91% and specificity of 89% in wheeze detection when compared to consensus assessment by a panel of pulmonary experts who performed auscultation of the same respiratory sounds [24]. Crackles were defined according to published criteria [26,27], and a "crackle counter" algorithm was developed analogous to one previously published by Murphy et al. [26]. To verify accuracy of the automatic crackle detector, all sound segments also underwent a manual auditory analysis by two pulmonologists (LB, SG), who were blinded to the results of the computerized wheeze and crackle quantification.

The adhesive pad with which the sensors were applied to the skin provided sound shielding. Environmental noise such as speech, ringing, beeps, bumps etc, were identified with an external microphone near the patient and electronically removed from the recording data. Occasional motion artifact and the baby's own crying interfered with the recording.

Lung sounds were recorded for a period of 5 minutes prior to treatment, and then at 10 and at 30 minutes afterwards, while the infant was relatively settled and not crying. Recording times varied somewhat, and were extended when necessary, to get enough "quiet breathing" data. To obtain adequate averaging of the acoustic data, we analysed breath segments that contained at least 5 consecutive interference-free breaths, for a total of 20 breaths. Wheeze Rate (percent of time wheezing of total breath time) [24] and crackle count (number of crackles per breath) were determined by the PulmoTrack^® ^for each breath cycle, and averaged over the 20 breaths. It usually took 4–6 hours per patient to edit and analyze the data.

### Statistical Analysis

Statistics were performed by a biostatistician (TN). A power calculation was not performed ahead of the study, since this was a pilot study intended mainly to test the feasibility and accuracy of automatic wheeze and crackle quantification in a population of very young infants, not intended to replace the clinical score, and with response to treatment being secondary.

Demographic and clinical history variables, numerical and categorical, were analyzed by Student t-test for independent samples and Fisher exact test, respectively. Clinical score and recorded acoustic breath sounds variables were analyzed by non-parametric tests: Mann-Whitney U test was used to compare between the groups, while Wilcoxon signed rank test was used to compare within-group differences over time. Relationship between clinical score and crackle or wheeze counts, was tested by Spearman correlation. P < 0.05 was considered as statistically significant.

## Results

Over the study period 87 patients with bronchiolitis were seen in the emergency department. Excluded were: 21 patients younger than 8 weeks, 22 seen on off hours when investigators were unavailable, 12 with other significant concurrent illnesses and 5 whose parents refused. Twenty-seven infants whose parents signed an informed consent (mean age 4.4 ± 0.8 months) were recruited, 12 received epinephrine and 15 albuterol. The groups were well balanced in terms of demographic and clinical parameters (Table 2). Pre-study treatment with either inhaled bronchodilators or corticosteroids was not different in the two groups.

**Table 2:** Demographic and clinical data of the sudy's 27 infants with RSV bronchiolitis.

**Study**	**Epinephrine**	**Albuterol**
Number	12	15
Age(months)	4.9 ± 0.8	4 ± 1.35
Gender(F/M)	4/8	4/11
Maternal smoking	5	7
Asthma in family	3	5
Atopy in family	3	2
Respiratory distress (days)	2.5 ± 0.48	2.2 ± 0.29
Poor feeding (days)	1.75 ± 0.44	2.2 ± 0.29
Fever (days)	1.41 ± 0.46	1.4 ± 0.32
Inh.Bronchodilators	3	2
Inh. Corticosteroids	1	1
Oral Corticosteroid	3	2

Poor feeding (not showing interest or eating significantly less (<75%) than normal) and Dyspnea (breathing fast, with effort and/or retractions) are according to parental history.

**Figure 1:**
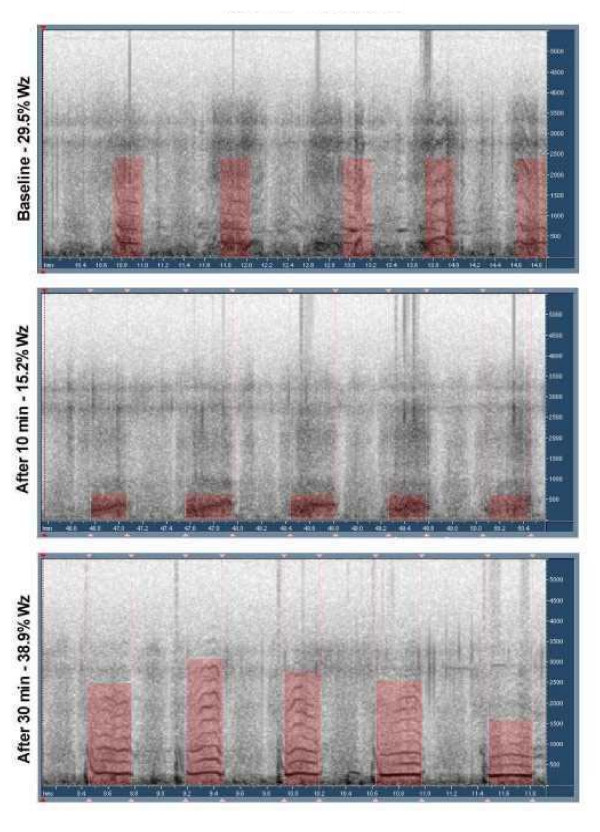
Sonogram of subject treated with albuterol, showing no response.

**Figure 2:**
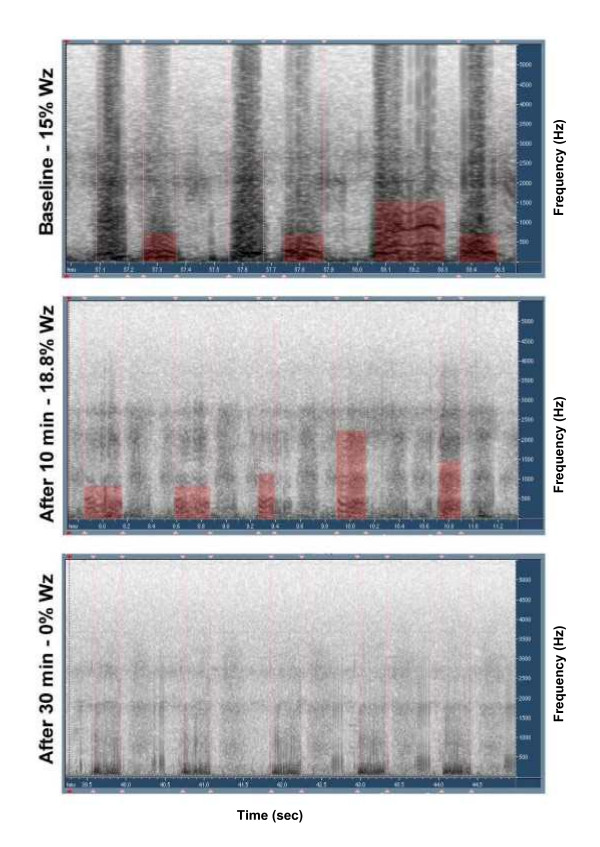
Sonogram of subject treated with epinephrine showing significant reduction in wheezing.

**Figure 3:**
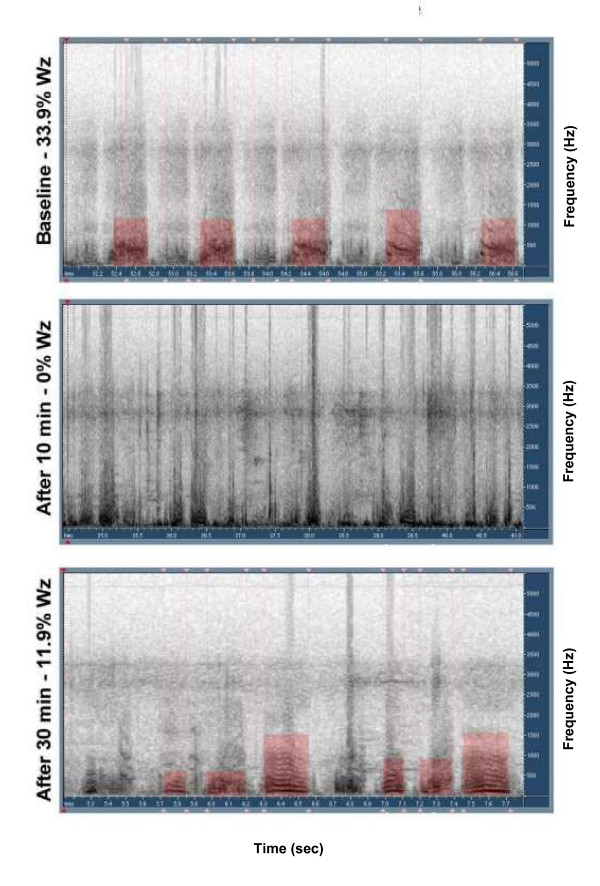
Sonogram of subject treated with epinephrine showing transient response with relapse.

Even though the crackle counter was not validated as part of this study, there was complete agreement between clinician and PulmoTrac results in all sound segments, in off-line auditory analysis of the data. No significant difference in wheezing and crackles by computerized lung sounds analysis was found between or within the groups prior to, 10 minutes post and 30 minutes post treatment. There was no significant change in objective quantification of wheezes and crackles between the groups or in the total clinical scores. Examples of acoustic wheeze detection in sonogram mode are shown in Figures 1, 2, 3. Figure 1 shows no response to albuterol, figure 2 demonstrates dramatic response to epinephrine with disappearance of wheezing, while figure 3 shows transient response with rebound wheezing at 30 minutes. Total clinical score and acoustic results for both treatment groups are summarized in Table 3.

**Table 3:** Results of the response to nebulized epinephrine and albuterol in respiratory, heart rate and total clinical scores, computerized wheeze count and computerized crackle count.

**Time**	**Epinephrine**	**Albuterol**	**p**
	Respiratory Rate Score*		
0	2.17 ± 0.27	2.4 ± 0.19	0.54
10	1.83 ± 0.3 §	2.67 ± 0.16	0.04
30	2.17 ± 0.34	2.47 ± 0.22	0.55
	Heart Rate Score*		
0	0.67 ± 0.22	1.0 ± 0.24	0.35
10	0.92 ± 0.23	1.2 ± 0.2	0.35
30	0.75 ± 0.13 §	1.4 ± 0.16	0.02
	Total Clinical Score*		
0	5.67 ± 0.71	7.27 ± 0.6	0.07
10	5.67 ± 0.83	7.13 ± 0.6	0.18
30	5.75 ± 0.77	7.47 ± 0.68	0.12
	Computerized Wheeze Rate*		
0	9.1 ± 3.4	5.5 ± 3.08	0.53
10	5.47 ± 3.26	9.11 ± 2.52	0.15
30	7.1 ± 3.63	11.9 ± 4.5	0.20
	Computerized Crackle Count*		
0	1.88 ± 0.59	1.74 ± 0.42	0.68
10	2.48 ± 0.92	1.14 ± 0.23	0.37
30	2.26 ± 0.7	1.31 ± 0.33	0.35

* Mean ± SD

§Significant result

Similar to the lung sounds, the total clinical score also did not differ significantly within or between the groups prior to, 10 minutes post and 30 minutes post treatment. In individual parameter analysis, a significant difference in favor of epinephrine was seen at 10 minutes in respiratory rate (p < 0.04), and at 30 minutes in heart rate (p < 0.02). There was no difference in oxygen saturation or respiratory distress score.

## Discussion

This pilot study evaluated the use of computerized wheeze and crackle quantification in assessing the acute response to either nebulized albuterol or epinephrine in infants with RSV-positive bronchiolitis. Although RSV Bronchiolitis is the most common cause of hospitalization for respiratory infection in infants, its therapy remains controversial. Therefore, objective measures for assessing response to treatment in RSV bronchiolitis are needed. Modern computerized acoustic lung sounds analysis technology now allow on-line quantification of wheezing and crackles, providing another objective tool for assessment of disease activity in infants with bronchiolitis. In this study, we demonstrated the feasibility of performing accurate objective acoustic measurements in assessing acute respiratory symptoms in young infants and the changes that occur with treatment. Further studies are needed to clarify its potential role as a clinical tool for assessing and following infants with acute respiratory illnesses.

The possible effectiveness of nebulized epinephrine for bronchiolitis, by reducing airway edema, was proposed 25 years ago [28] by Wohl and Chernick. Since then, many studies have evaluated the effect of nebulized epinephrine vs. albuterol and/or placebo in infants with bronchiolitis. While some reported an improved effect of epinephrine, others showed no, or even deleterious, effect [14-20]. Overall, it is difficult to compare the various studies, due to their different dose schedules, populations and outcome measures. The recent Cochrane Review on the use of bronchodilators in bronchiolitis [29] concluded that there is no strong evidence for their benefit, either short or long term. Similar to these previous RSV studies, our study indicates that there is no significant overall difference in the short-term effect of nebulized epinephrine compared to albuterol in these infants, when assessed by both objective acoustic analysis of wheezing and crackles and a combined clinical score. However, minor differences in heart and respiratory rates in favour of epinephrine were found, the clinical significance of which is uncertain.

Crackles and wheezing are the cardinal auscultatory findings in bronchiolitis. In asthma, the degree of wheezing measured as Tw/Ttot has been shown to correlate with severity of airway obstruction [30]. Tal et al. [23] studied 16 infants with bronchiolitis and wheezing, of whom 7 responded to albuterol. However, his study population consisted of older infants (mean age 9.4 vs. 4.4 months), the majority of whom had either previous episodes of wheezing, family history of asthma or atopy or bronchopulmonary dysplasia. They were therefore at higher risk of wheezing with viral infections or other triggers. In bronchiolitis, due to distal airway edema and secretions, crackles are often produced. Young babies with RSV bronchiolitis characteristically have more crackles, explaining the difference in wheezing between the two patient populations. Reduction of edema and drying of secretions by epinephrine might be expected to result in reduced crackles. Modern computerized acoustic lung sounds analysis technology (mainly due to fast high-RAM computers) now allow rapid accurate quantification of wheezing and crackles, providing an objective tool for assessment of disease activity in infants with bronchiolitis. In our study to evaluate this tool, we found no overall short-term advantage of nebulized epinephrine compared to albuterol as assessed by computerized wheeze and crackle quantification and by total clinical score. However, it should be noted that the sample size in this pilot study was designed primarily to evaluate the acoustic method and therefore does have adequate power to detect clinical response. A larger study is necessary to assess the correlation between the computerized crackle and wheeze counts and the Clinical Score in response to treatment in RSV bronchiolitis.

## Conclusion

Computerized lung sound analysis is feasible in young infants with RSV bronchiolitis and provides a non-invasive, quantitative measure of wheezing and crackles. In a pilot study using computerized lung sound analysis, we could not demonstrate any significant overall short-term effect of either nebulized epinephrine or albuterol, nor a difference between them.

## Competing interests

The author(s) declare that they have no competing interests.

## Authors' contributions

RB – Design of the study, drafting and revising the manuscript

NE – recruited the patients and was the responsible physician for their care

SS – performed the study procedures

NT – statistical support and statistical analysis of the data

GT – technical assistance in lung sounds recording and analysis of the data

SG – review and analysis of the data

LB – Design of the study, quality assurance, analysis, drafting and revising the manuscript

## Pre-publication history

The pre-publication history for this paper can be accessed here:


